# Enhancing healthcare efficiency: leveraging advanced maintenance management for optimal staff performance

**DOI:** 10.1108/JHOM-03-2025-0134

**Published:** 2025-09-09

**Authors:** Soultana (Tania) Kapiki, Anthitsa Pappa

**Affiliations:** Department of Organisation Management, Marketing and Tourism, International Hellenic University, Thessaloniki, Greece; University General Hospital of Ioannina, Ioannina, Greece

**Keywords:** Staff performance, Healthcare maintenance management, CMMS, Artificial intelligence (AI), Internet of Things (IoT), Blockchain, SMART-Maintenance Framework (SMF), Work environment, Employee satisfaction, Leadership in healthcare, Healthcare efficiency, Operational optimisation

## Abstract

**Purpose:**

This study explores how effective maintenance strategies enhance healthcare staff performance. It evaluates their influence on the work environment and operational efficiency, examines the benefits of Computerised Maintenance Management Systems (CMMS), artificial intelligence (AI), the Internet of Things (IoT) and blockchain and highlights the role of leadership in adopting these innovations. Real-world examples illustrate their success in boosting workforce productivity and overall healthcare efficiency.

**Design/methodology/approach:**

An online survey was conducted among 102 healthcare professionals in Greece, including doctors, nurses, administrative staff and technicians. The study applies ANOVA, Pearson correlation and comparative analysis to examine maintenance management and staff performance, supported by a literature review on maintenance benefits, leadership and the utilisation of emerging technologies. Findings are contextualised through cross-role and unit-level comparisons.

**Findings:**

Results underscore the importance of a well-maintained work environment in boosting staff satisfaction and performance. Factors such as lighting, air quality and infrastructure were linked to reduced stress and improved job satisfaction. The study emphasises the benefits of emerging technologies in improving efficiency and identifies gaps in training and adaptation to modern requirements. Leadership plays a pivotal role, with findings indicating the need for better communication and prompt issue resolution.

**Research limitations/implications:**

While offering valuable insights, this study has several limitations. Convenience sampling via hospital websites and social media may introduce selection bias, as digitally active respondents could differ from others, limiting generalisability. A small sample size (*n* = 102) further restricts representativeness across the healthcare workforce. Self-reported data may also skew results due to recall and social desirability biases. To strengthen reliability, future research should incorporate objective data sources and qualitative methods like interviews. Additionally, the SMART-Maintenance Framework, though promising, remains conceptual. Validation through pilot and longitudinal studies is essential to determine its long-term impact on healthcare maintenance and staff well-being.

**Practical implications:**

This research offers the following actionable and easily adoptable practical solutions: (a) The SMART-Maintenance Framework (SMF): a structured framework for integrating technology and management best practices, helping healthcare organisations optimise maintenance operations and ultimately improve patient care; (b) Strategic guidelines for adopting the SMF effectively; (c) Technology-driven solutions about emerging technologies’ integration: implementing CMMS, AI, IoT and blockchain, healthcare administrators can streamline maintenance processes, minimise equipment downtime and improve overall working conditions and (d) Key recommendations for healthcare staff and administrators.

**Originality/value:**

This research provides actionable recommendations, addressing key challenges and leveraging technological solutions to create a more efficient workplace. It also introduces the SMART-Maintenance Framework (SMF), an innovative model integrating digital tools and management insights to enhance healthcare maintenance and staff performance. The findings contribute valuable insights to optimising healthcare delivery.

## 1. Introduction

In the dynamic landscape of healthcare, effective maintenance management is pivotal to the smooth operation of health units. It ensures the reliability of medical equipment, maintains a safe working environment, and supports high-quality patient care. As healthcare services evolve to meet rising demands, the need for robust maintenance strategies becomes even more essential.

This study examines the critical role of effective maintenance management in optimising healthcare staff performance assessing its impact on the working environment and staff performance, exploring the benefits of Computerised Maintenance Management Systems (CMMS), Artificial Intelligence (AI), Internet of Things (IoT), and Blockchain Technology, and evaluating the role of leadership in maintenance management.

Recent studies highlight the importance of maintenance management in healthcare settings. For example, Krijgsheld *et al.* (2022) emphasise its role in job performance, while Pomaranik and Kludacz-Alessandri (2024) discuss its impact on employee outcomes in the public healthcare sector. Together, these studies underscore the need for a well-maintained work environment to enhance healthcare staff performance.

Greek health units face unique challenges, including economic constraints and rapid technological change, which lead to outdated infrastructure and insufficient training, ultimately affecting staff performance and workplace conditions. Understanding current practices and their effect on staff is crucial for improving healthcare delivery.

International case studies further illustrate the value of advanced maintenance technologies. At Riddle Memorial Hospital (USA), CMMS reduced service calls and boosted staff efficiency. At the Health and Hospital Corporation in Indiana (USA), a centralised CMMS improved compliance and resource allocation. At the Area 25 Health Centre in Lilongwe (Malawi), AI-enabled fetal monitoring reduced neonatal deaths and provided staff with real-time insights. These examples show how technology enhances performance, safety, and operational effectiveness.

Comparisons between healthcare units using advanced maintenance systems and those relying on reactive strategies reveal stark differences. Units adopting CMMS and AI-driven maintenance report higher staff satisfaction, fewer technical failures, and lower incident rates, underscoring the necessity of adopting these technologies across all health units. In contrast, facilities relying on reactive maintenance strategies often experience prolonged downtimes and operational inefficiencies, further impacting staff performance and patient care quality (Open Medscience, 2025).

Maintenance management also intersects with patient safety and operational efficiency. Integrating these domains provides a comprehensive understanding of how maintenance influences performance, ensuring not only reliable equipment but also a safer and more efficient healthcare environment (Yousefli *et al.*, 2017).

To tackle the complexity of maintenance management in healthcare, this study introduces the SMART-Maintenance Framework (SMF), an integrated approach that combines digital tools (CMMS, AI, IoT, and Blockchain) with leadership practices to enhance maintenance processes and staff performance. Unlike broad models such as Total Productive Maintenance (TPM) or ISO 41001, which offer generic guidance for facility or manufacturing environments, the authors tailored SMF specifically for healthcare settings, addressing operational stressors and the unique needs of healthcare personnel.

The SMF is conceptually grounded in key organisational behaviour and facility management theories. Socio-Technical Systems Theory supports the integration of digital tools with human-centred leadership to optimise outcomes in complex healthcare settings. Organisational Support Theory further explains how managerial responsiveness and communication practices influence employee satisfaction and performance. Additionally, the framework aligns with the Resource-Based View by positioning well-maintained infrastructure and trained personnel as strategic assets.

The SMF follows a logic model structure, where five core domains (environmental conditions, equipment reliability, training readiness, technology adoption, and leadership effectiveness) serve as inputs. These drive activities such as digital tool integration, Key Performance Indicators (KPI) tracking, and communication improvement, leading to outputs like reduced stress and improved coordination. The final outcomes include enhanced staff satisfaction, performance, and ultimately, patient safety. This theoretical and operational alignment strengthens the SMF’s utility and prepares it for empirical validation through pilot or longitudinal studies in future research.

This study addresses the following research questions:

RQ1.
What is the impact of effective maintenance management on the work environment and staff performance in health units?

RQ2.
What are the benefits of using CMMS and other emerging technologies like AI, IoT, and Blockchain?

RQ3.
What role do leadership and responsibilities play in the effectiveness of maintenance management?

The research aims to fill gaps in the literature by providing insights into the maintenance management practices in health units and their impact on healthcare staff performance. The findings could influence policy and management strategies, leading to improved maintenance protocols, enhanced working environments, and better overall healthcare outcomes. By implementing advanced technological solutions and the proposed SMF, healthcare organisations can move towards a more efficient, technology-driven approach to facility management, ultimately benefiting both staff and patients.

## 2. Literature review

The performance of healthcare staff is vital to the effectiveness and efficiency of healthcare organisations. Effective maintenance management fosters a supportive work environment, influencing staff performance. This literature review explores existing research on maintenance management, working environments, and healthcare staff performance, highlighting their interconnections.

### 2.1 Advantages of effective maintenance management

Effective maintenance management improves medical facility efficiency by optimising space, ensuring equipment reliability, and maintaining safety and cleanliness. Prioritising patient care, healthcare facilities must meet safety and hygiene standards. Automated metering provides real-time alerts on electricity use, enabling timely interventions and energy savings. Preventive maintenance of emergency lighting ensures functionality during crises (Fatima *et al.*, 2018). Effective maintenance reduces downtime, boosts patient satisfaction, minimises disruptions, and ensures seamless healthcare delivery (Yousefli *et al.*, 2017). Shohet and Lavy (2004) underscore the need for structured maintenance departments to ensure reliability and efficiency. Specialised teams are essential for addressing healthcare-specific challenges.

Beyond operational efficiency, effective maintenance yields cost savings. Preventing negligence-related incidents avoids legal liabilities. Proper strategies extend equipment lifespan, reduce breakdowns, and lower repair costs (Kelvin-Agwu *et al.*, 2024). Dhillon (2002) supports proactive maintenance for cost savings. Kaplan and Porter (2011) link efficient maintenance to reduced operational costs and better resource use.

Global benchmarks, such as those set by the World Health Organization (2015), emphasise preventive maintenance, staff safety, and system reliability as foundational principles, reinforcing the need for structured frameworks like SMF in both developed and resource-constrained healthcare systems.

### 2.2 Leadership and responsibilities in maintenance management

Facility management coordinates activities for efficient operation, including building design, space planning, energy, safety, and maintenance (Cotts *et al.*, 2009). Maintenance management focuses on equipment and infrastructure upkeep (Pappa and Kapiki, 2022), including preventive and corrective maintenance, repairs, and reliability (Wireman, 2005). Facility managers oversee design, maintenance, and safety, requiring rigorous training. Improving equipment reliability involves collaboration with suppliers and service providers (American Hospital Association, 2022). They also manage equipment procurement and renovations (Shohet and Lavy, 2004). Yousefli *et al.* (2017) highlight the importance of training and external collaboration in enhancing maintenance practices.

### 2.3 Healthcare staff performance

Staff performance includes task, contextual, adaptive, and counterproductive behaviours (Griffin *et al.*, 2017; Borman and Motowidlo, 1997). Task performance covers core job duties, while contextual performance supports the organisational environment. Adaptive performance is key in dynamic healthcare settings, whereas counterproductive behaviour harms the organisation. Buljac-Samardzic *et al.* (2020) and Krijgsheld *et al.* (2022) emphasise team-based approaches, training, and supportive environments to improve performance.

The relationship between maintenance management and staff performance in healthcare can be conceptually framed as a sequential pathway: Maintenance Management → Work Environment → Staff Stress and Satisfaction → Staff Performance. Effective maintenance practices shape the physical and organisational work environment, which in turn influences the psychological well-being and satisfaction of healthcare staff. These factors directly impact overall staff performance. This conceptual pathway is supported by key theoretical foundations: Socio-Technical Systems Theory, which highlights the need to jointly optimise technical systems and human interactions; Organizational Support Theory, which links management responsiveness to employee outcomes; and the Resource-Based View, which considers trained personnel and well-maintained infrastructure as strategic assets contributing to organisational success.

Burnout remains a critical consequence of unmanaged work-related stress, particularly in healthcare environments. Maslach and Leiter (2016) identify key drivers of burnout, including lack of control, insufficient recognition, and poor leadership. These elements are closely tied to the domains addressed by the SMF, such as leadership responsiveness, environmental stressors, and workload management.

### 2.4 Advanced maintenance management technologies

This section highlights how CMMS, AI, IoT, and Blockchain collectively contribute to more proactive, transparent, and staff-supportive maintenance environments in healthcare.

Sustainable healthcare equipment is essential, as frequent failures can severely impact patients and disrupt operations. The COVID-19 pandemic underscored the importance of reliability and proactive strategies. CMMS is among the most widely used tools in this area, scheduling, tracking, and documenting tasks to enhance reliability and reduce downtime. It also supports predictive analytics, data-driven decision-making, and long-term asset planning (Tsang, 1995; Mobley, 2002).

In hospital settings, maintenance is often fragmented, poorly documented, and hindered by information overload. Technologies such as CMMS, EDMS, EMS, and BAS improve documentation and efficiency (Alhurayess and Darwish, 2012; Wong *et al.*, 2005), but their isolated use can create silos. Integrating these systems enables cost savings, faster communication, and improved safety. Becerik-Gerber *et al.* (2011) emphasise the need for seamless information flow from planning to operations, while BIM-based coordination has shown positive results in facility change management (Sebastian, 2011).

Beyond CMMS, emerging technologies like AI, IoT, and Blockchain are reshaping healthcare maintenance ecosystems. AI enables predictive analytics and resource optimisation (Duigou, 2023; Kaur, 2024), IoT facilitates real-time condition monitoring (Shamayleh *et al.*, 2020; Innovent.io, 2023), and Blockchain enhances the security and transparency of records (Kasyapa and Vanmathi, 2024; HyScaler, 2025). It also fosters collaboration among stakeholders, promoting trust and shared data access (Laturkar and Khaiyum, 2023).

Strategically integrating these technologies addresses fragmentation and improves operational efficiency. IoT monitors equipment continuously, AI anticipates failures, and Blockchain ensures traceability (Mehta, 2025). Together, they form the digital foundation of the SMF, enabling data-informed decisions and supporting staff through reduced stress and improved system reliability.

To strengthen digital tool implementation, the SMF aligns with the Unified Theory of Acceptance and Use of Technology (UTAUT) proposed by Venkatesh *et al.* (2003), which highlights performance expectancy, effort expectancy, and social influence as key factors affecting adoption. These elements are implicitly addressed in the SMF’s emphasis on training, leadership, and ease of digital integration.

### 2.5 Interconnections and impact

Adopting advanced maintenance management practices and leveraging emerging technologies enhance operational efficiency, patient safety, and staff satisfaction. These improvements ultimately lead to better healthcare outcomes. Effective maintenance ensures medical equipment and facilities remain in optimal condition, directly influencing the work environment. A well-maintained setting reduces stress, increases job satisfaction, enhances healthcare staff performance, and promotes safety and efficiency (Krijgsheld *et al.*, 2022; Pomaranik and Kludacz-Alessandri, 2024). Mahfoud *et al.* (2018) highlight the importance of continuous training and technological advancements in fostering a productive workplace.

The integration of CMMS and emerging technologies has transformed maintenance activities, enabling real-time monitoring, predictive maintenance, and efficient work order management. AI-driven predictive maintenance minimises equipment downtime (Kaur, 2024), IoT facilitates continuous monitoring and proactive interventions (Shamayleh *et al.*, 2020), and Blockchain enhances data security and interoperability (Kasyapa and Vanmathi, 2024). Comparative studies indicate that healthcare units utilising advanced maintenance systems report higher staff satisfaction and lower incident rates, reinforcing the necessity of widespread adoption (Hamed *et al.*, 2025).

The proposed SMART-Maintenance Framework also aligns with the principles of the Quadruple Aim in healthcare, which seeks to improve the patient experience, enhance population health, reduce healthcare costs, and support healthcare staff well-being. By improving the working environment and reducing maintenance-related stress through effective and technology-supported practices, the framework contributes not only to internal efficiency but also to broader healthcare system goals.

## 3. Methodology

To explore the research questions and evaluate the proposed framework, the study employed a mixed-methods research design.

### 3.1 Research design

This study adopts a quantitative research design to evaluate the impact of advanced maintenance management practices on the working environment and performance of healthcare staff in Greek health units. The cross-sectional survey approach allows for the collection and analysis of data at a single point in time, providing a snapshot of the current state of maintenance management and its effects on staff performance. Additionally, the study integrates real-world examples to illustrate the practical benefits of advanced maintenance technologies such as CMMS, AI, IoT, and Blockchain.

The authors selected survey items and thematic areas based on three theoretical frameworks: Socio-Technical Systems Theory informed the inclusion of items relating to digital tools and staff–technology interactions; Organizational Support Theory shaped questions on leadership responsiveness and communication; and the Resource-Based View justified the focus on staff training and well-maintained infrastructure as strategic assets. Although this study remains exploratory, these theories provided conceptual grounding in the design and analysis.

### 3.2 Participants

The sample consists of 102 healthcare professionals from various public and private health units across Greece. Participants include doctors, nurses, administrative staff, and technicians. The respondents are predominantly female, and their ages range from under 25 to over 56 years, with the largest group being those aged 46–55 years.

### 3.3 Sampling method

The authors used a convenience sampling technique, distributing invitations through hospital websites, professional networks, and online healthcare groups. The goal was to achieve a diverse representation of healthcare staff across different roles and employment types, including permanent and non-permanent positions. This approach may introduce limitations regarding the generalisability of the findings, as convenience sampling does not ensure a representative sample. Moreover, the use of self-reported questionnaires may result in response bias, including social desirability effects.

### 3.4 Data collection

The authors collected data using an online questionnaire developed in Google Forms. They adapted the questionnaire from the “Homogeneous Employee Subjective Assessment Questionnaire” by ELINYAE (Hellenic Institute for Occupational Health and Safety), incorporating additional questions relevant to advanced maintenance management and working environment conditions. The questionnaire consisted of five sections with a total of 57 questions, covering:

1. Demographic Data: Gender, age, marital status, level of education, job roles, type of employment, and years of service.
2. Working Environment: Building structures, environmental conditions, use of computers and medical equipment, and training received.
3. Maintenance Management: Adaptability to technological advancements, role of management, and efficiency of maintenance practices.
4. Stress Factors: Sources of work-related stress, including equipment failures, outdated technology, and workplace design.
5. Technological Integration: Use of computerised maintenance systems, AI, IoT, Blockchain, and preferences for technological upgrades.

Subsequently, the authors conducted a pilot test of the questionnaire with 12 healthcare professionals, including two doctors, three nurses, two administrators, two laboratory staff, and three maintenance personnel. Based on their feedback, minor adjustments were made to clarify terminology and improve the structure of items related to emerging technologies. This ensured relevance and comprehension in the Greek healthcare context.

### 3.5 Data analysis

The authors analysed responses using SPSS (Statistical Package for the Social Sciences). Descriptive statistics (frequencies, percentages, and visual summaries) were used to identify patterns in maintenance practices, working conditions, and their effects on employee performance and satisfaction.

Internal consistency was confirmed with Cronbach’s Alpha (α = 0.88) for items related to maintenance management and interdepartmental coordination, indicating excellent reliability. Construct validity was supported through Exploratory Factor Analysis (EFA), with the Scree Plot suggesting a single-factor solution.

To assess relationships among key variables, the authors applied ANOVA, identifying significant group differences based on job roles and workplace perceptions. While the analysis remained exploratory, future research could include regression or mediation models to strengthen causal inferences.

The authors also conducted a Pearson correlation analysis to explore how variables such as equipment reliability, leadership, training, and stress interact. The results, presented in Appendix A (Table A2), offer additional insight into the strength and direction of these associations.

Finally, a comparative analysis across healthcare units highlighted key differences in maintenance efficiency, environmental conditions, technology integration, and leadership practices.

While the analysis relied on self-reported data, future research could strengthen validity by triangulating survey results with maintenance logs, incident reports, or digital system records.

### 3.6 Ethical considerations

Participation in the study was voluntary, and informed consent was obtained from all respondents. Confidentiality and anonymity were maintained throughout the study, ensuring that individual responses could not be traced back to participants. Approval for this study was granted by the appropriate Institutional Board and permission was received from ELINYAE for the use of their questionnaire.

## 4. Findings and discussion

Building on the methodology described above, the following section presents the study’s key findings.

### 4.1 Demographic data

The survey sample includes 102 respondents, predominantly women. The largest age group is 46–55 years, followed by 36–45 years, with smaller percentages in other age groups. Most participants work in public health units, with a smaller share in private clinics and other facilities such as health centres. The sample primarily consists of nursing and midwifery staff, followed by medical, administrative, laboratory, and other healthcare personnel. Most respondents hold permanent positions, while a smaller portion are in non-permanent roles. The largest group has 11–20 years of service, followed by those with 21–30 years, fewer than 10 years, and more than 31 years.

**Figure 1:**
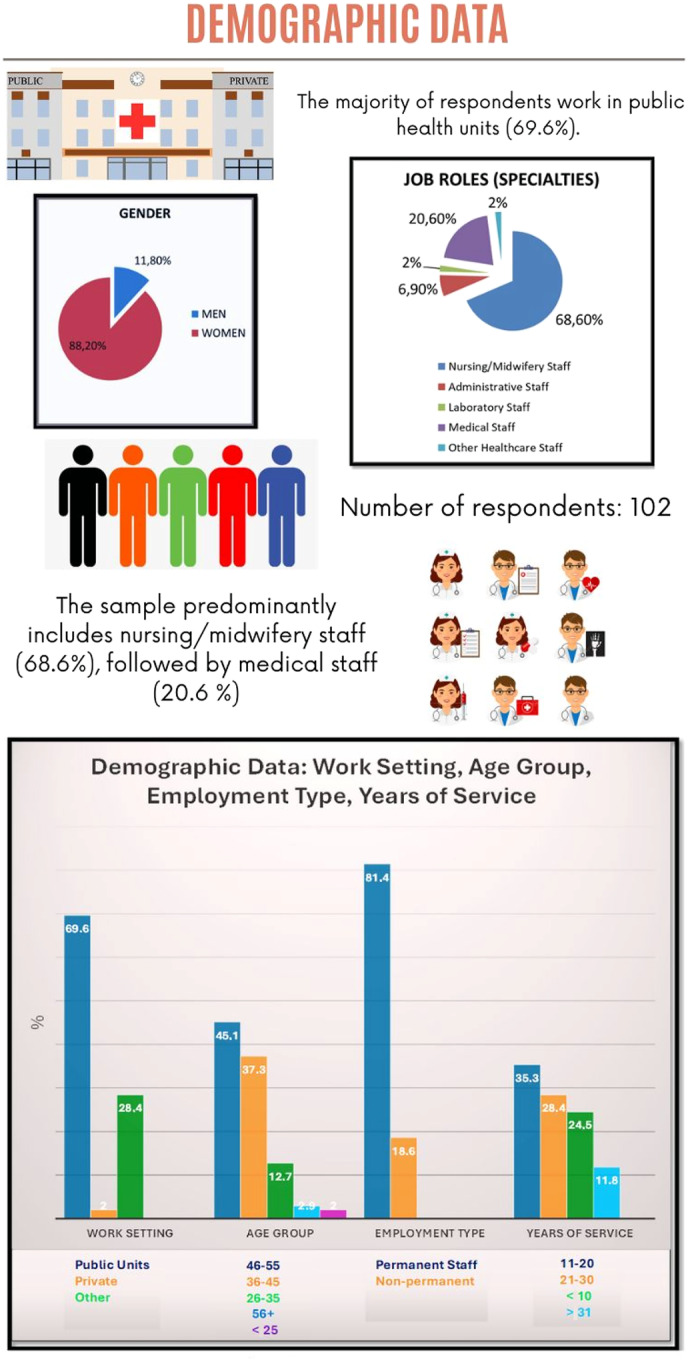
Demographic data of respondents. Source: Authors’ own work

See Figure 1 for a visual summary of the findings.

### 4.2 Impact of work environment and maintenance management on employee performance in healthcare units

#### 4.2.1 Satisfaction and challenges in the workplace environment

The study reveals a mixed picture of workplace satisfaction. Nearly half of respondents are content with their department’s building structure, and most rate lighting and ventilation positively. However, many report physical obstacles and risks such as falling materials. While noise levels are mostly deemed normal, a notable portion considers them high. Environmental satisfaction varies, with generally positive feedback on winter temperatures, and summer humidity.

Identifying environmental factors that influence employee performance helps guide improvements. These include (a) vibrations, temperature, ventilation, humidity, (b) lighting, (c) noise, and (d) physical infrastructure.

The authors conducted an ANOVA analysis to examine how workplace and organisational factors affect staff performance. The results indicate:

1. Air quality and timely issue resolution significantly influence staff perceptions of maintenance delays.
2. Management responsiveness is critical to overall maintenance effectiveness.

These findings underscore the importance of a well-maintained environment and prompt maintenance in improving staff satisfaction and performance in healthcare settings.

#### 4.2.2 Causes of stress

Staff report several stressors linked to poor maintenance management and communication. Major factors include failures in technological equipment, outdated tools, and inadequate workplace design. Additional stress arises from the lack of modern equipment and insufficient communication between maintenance teams and staff. When asked whether they would recommend their workplace to others, only a small portion responded positively, with most being partially positive and a minority negative.

#### 4.2.3 Equipment handling

A significant portion of respondents use computers and operate medical equipment at work. However, fewer than half have received appropriate training. Maintenance delays are a common burden, highlighting the need for improved planning and support.

**Figure 2:**
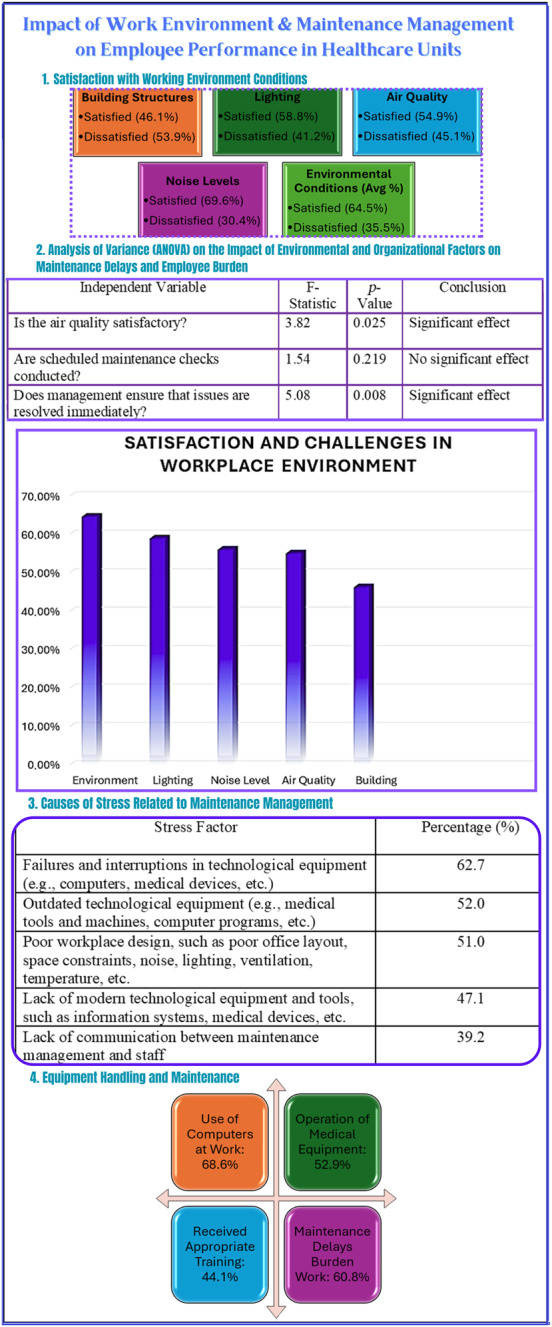
Impact of work environment and maintenance management on employee performance in healthcare units. Source: Authors’ own work

Figure 2 presents an infographic summarising these findings.

### 4.3 Leveraging advanced technologies for effective maintenance management in healthcare facilities

#### 4.3.1 The need for technological advancement in healthcare maintenance

Healthcare employees recognise the need for technological upgrades and environmental sustainability. Most respondents support the replacement of outdated equipment and improvements in building energy efficiency. However, many note a lack of action in these areas. A significant portion question maintenance management’s ability to meet modern requirements, and fewer than half report receiving organised training on new technologies.

#### 4.3.2 Transforming maintenance management with CMMS and emerging technologies

The integration of advanced technologies is essential for effective maintenance in healthcare units. Survey results indicate:

1. Widespread use of computerised scheduling, though manual recording remains common.
2. Partial satisfaction with manual systems for organisation and tracking.
3. Strong preference for computerised/advanced systems, viewed as more efficient.

These findings suggest that technological tools can enhance daily operations and long-term maintenance of infrastructure and medical equipment.

To statistically validate perceived maintenance burden across roles, the authors conducted an ANOVA. Results showed a significant difference (*F* = 2.88, *p* = 0.027, η^2^ = 0.106), with doctors reporting higher burden than nurses (mean: 2.86 vs. 2.54; *p* = 0.0046).

Additional analysis explored how workplace and system factors influence maintenance perceptions. Findings revealed:

1. Rapid management response significantly reduces perceived delays.
2. Computerised systems lower staff burden but have limited impact on perceptions of overall organisation.
3. The strongest predictor of perceived maintenance effectiveness is whether administration resolves issues promptly (*F* = 3.93, *p* = 0.023).

In summary, advanced systems reduce burden, while leadership responsiveness remains critical to improving perceptions of maintenance organisation.

**Figure 3:**
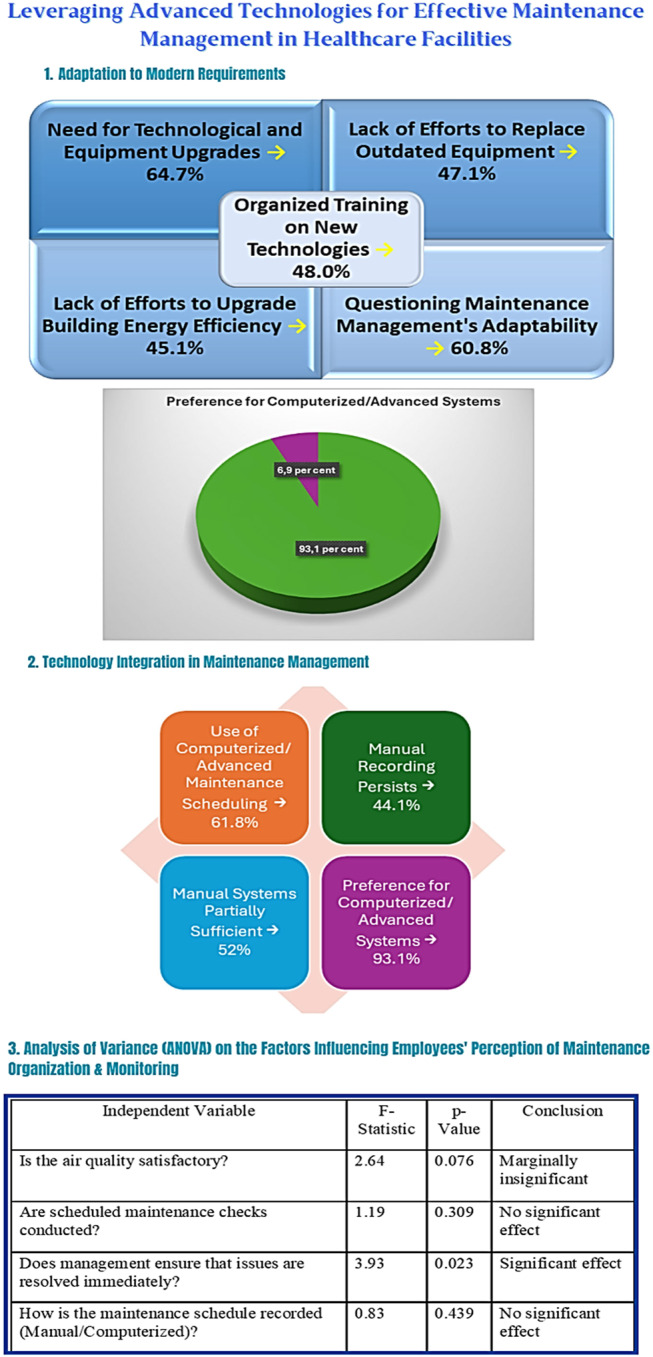
Leveraging advanced technologies for effective maintenance management in healthcare facilities. Source: Authors’ own work

See Figure 3 for a visual summary of these findings.

#### 4.3.3 International applications of advanced maintenance technologies

Globally, the integration of advanced technologies such as CMMS, AI, IoT, and Blockchain into healthcare maintenance management has led to measurable improvements in staff performance and operational efficiency. The following real-world examples illustrate successful implementation:

1. Hospital “Le Scotte” Siena, Italy: Introduced IoT, robotics, and AI to enhance safety, productivity, and quality. The initiative improved maintenance workflows, reduced equipment downtime, and increased staff efficiency (Luschi *et al.*, 2024).
2. Sheba Medical Centre, Israel: Integrated AI and IoT for predictive maintenance and real-time equipment monitoring, reducing downtime and improving staff productivity.
3. Mayo Clinic, USA: Applied AI and robotics to streamline maintenance workflows and ensure equipment reliability, resulting in fewer failures and improved patient care.
4. Singapore General Hospital, Singapore: Deployed IoT sensors and AI algorithms to optimise maintenance, reduce downtime, and enhance staff efficiency.

### 4.4 The role of management in the efficiency and reliability of maintenance

Survey responses highlight several areas where management significantly influences maintenance outcomes:

1. Scheduled maintenance: While some respondents report regular checks, many experience irregular or absent scheduling, indicating a need for better planning.
2. Problem resolution: Many believe that identified problems are not corrected promptly, with some stating that management does not ensure immediate correction of issues during audits.
3. Safety vs. cost: Half of the participants believe safety is only partially prioritised over cost.
4. Confidence in administration: There is partial confidence in administration’s ability to handle maintenance effectively and manage safety issues.
5. Communication: Despite some concerns, most acknowledge relatively effective communication between maintenance and other departments.
6. Training seminars: Many express a desire for more training on equipment, which is currently lacking.

The impact of ineffective maintenance is substantial, with many participants reporting that delays burden their daily work.

Below are two examples that further illustrate how leadership affects the efficiency and reliability of hospital operations, in Greece:

1. Greek National Health System (NHS): A study on public hospital efficiency found that effective management and strategic planning (particularly team coordination and professional standards) significantly improve operational performance (Mitropoulos *et al.*, 2013).
2. Greek NHS Hospitals: Research on hospital managers’ involvement in operational planning revealed that strategic planning and leadership are critical for achieving financial and operational objectives. Factors such as service evaluation and staff engagement were found to have the highest impact on hospital efficiency (Letsios *et al.*, 2022).

### 4.5 Comparative insights and innovative contributions

To contextualise the main findings, this section presents a comparative analysis with relevant studies, highlighting the unique contributions of the current research.

#### 4.5.1 Environmental conditions and staff performance

The research uniquely identifies specific environmental challenges, such as high winter humidity and elevated noise levels, which are particularly relevant to Greek health units. This detailed identification of environmental factors provides actionable insights for targeted improvements in the working environment. In contrast, Ntshebe *et al.* (2022) found that a safe and functional workspace positively influences staff performance in South Africa but did not elaborate on specific environmental stressors. The current study’s granular detail (e.g. falling objects, spatial congestion) provides a more operationally useful perspective for targeted interventions.

#### 4.5.2 Equipment failures as a primary stressor

Technological equipment failures and outdated devices emerge as major stressors among healthcare staff. While Ntshebe *et al*. (2022) linked poor maintenance to stress and reduced performance, the present study goes further by identifying equipment unreliability as a measurable source of stress. This emphasis on the emotional and operational impact of outdated technology is less visible in prior studies and points to the need for more frequent equipment updates and standardised maintenance protocols.

#### 4.5.3 Training gaps and maintenance delays

Fewer than half of respondents report receiving proper training on maintenance systems. The lack of training is statistically linked to maintenance delays and reduced operational efficiency. While Beniacoub *et al.* (2021) emphasised the importance of training in CMMS implementation, the current study provides quantitative evidence of how undertraining disrupts healthcare operations. This level of quantification is often missing from related studies and highlights a novel, evidence-based argument for investing in targeted training programs.

#### 4.5.4 Technological readiness and organisational culture

Staff responses reveal a strong readiness to embrace advanced maintenance technologies such as CMMS, AI, IoT, and Blockchain. While many studies (e.g. Link to the website) advocate for these tools, few provide empirical insights into staff attitudes toward adoption. The current findings suggest a positive organisational culture toward innovation, which healthcare leaders can leverage to support digital transformation and sustained implementation of advanced systems in Greek healthcare settings.

#### 4.5.5 Leadership gaps and staff trust

Survey data point to specific leadership weaknesses, particularly in timely issue resolution and trust-building communication. Staff perceptions quantitatively support these gaps, revealing a broader lack of administration responsiveness. While previous research (e.g. Link to the website) highlights the general importance of leadership, this study offers operational detail on what is lacking. These insights form a practical roadmap for improving administration effectiveness, accelerating issue resolution, and supporting staff morale.

Comparative analysis confirms a shared understanding of the positive impact that effective maintenance management and digital adoption have on staff performance and healthcare efficiency. While this study focuses on the Greek healthcare context, findings from other studies reinforce the broader applicability of advanced maintenance practices across diverse systems.

Although the statistical analysis was primarily exploratory and comparative, emerging patterns offer valuable theoretical insights. The alignment of environmental conditions, equipment reliability, training access, and managerial responsiveness with staff stress and satisfaction supports the SMART-Maintenance Framework’s core structure. These results also affirm key assumptions from Socio-Technical Systems Theory, and Organizational Support Theory, which links perceived managerial support to morale and performance. This theoretical alignment strengthens the framework’s academic contribution and lays the groundwork for future empirical validation.

### 4.6 Summary of findings mapped to SMF

The findings map directly to the five domains of the SMART-Maintenance Framework (SMF). Environmental stressors such as excessive noise and humidity align with the SMF’s environmental tracker. Equipment-related issues, including outdated or malfunctioning technology, significantly contribute to staff stress and dissatisfaction. Limited training access hinders timely issue resolution, confirming the importance of structured upskilling. Strong staff support for digital systems like CMMS, AI, and IoT indicates high technological readiness. Finally, delays in problem resolution and low trust in administration highlight persistent leadership and communication challenges.

Together, these insights affirm the SMF’s value in healthcare. They also reinforce the theoretical foundations of the framework: Socio-Technical Systems Theory supports its integration of environmental and technological factors, and Organizational Support Theory aligns with SMF’s emphasis on leadership and responsiveness, illustrating how trust and satisfaction are shaped by managerial action and underpin sustained staff engagement.

## 5. Practical implications and strategic guidelines

Based on the study’s findings, the authors propose an innovative framework that integrates technology and management insights to improve healthcare maintenance.

### 5.1 The SMART-Maintenance Framework (SMF)

The SMF integrates key research findings with a simple digital tool (dashboard) to enhance maintenance processes in healthcare settings. It stands out because it:

1. Combines management insights with a user-friendly digital tool, requiring no complex programming or advanced analytics.
2. Responds directly to identified gaps, including staff stress, outdated equipment, training needs, and managerial challenges.
3. Adapts to different healthcare units (e.g. public vs. private hospitals, varying department needs).
4. Integrates easily with existing hospital maintenance systems.

The following approach outlines how hospitals can implement the SMF effectively.

### 5.2 Framework template for data collection

To make SMF effective, hospitals need to track maintenance-related issues, training, technology adoption, and leadership efficiency. Below is a data collection framework that hospitals can use in Google Sheets, Excel, or a simple digital tool (see Table 1).

**Table 1:** SMART-Maintenance Framework (SMF): data collection template

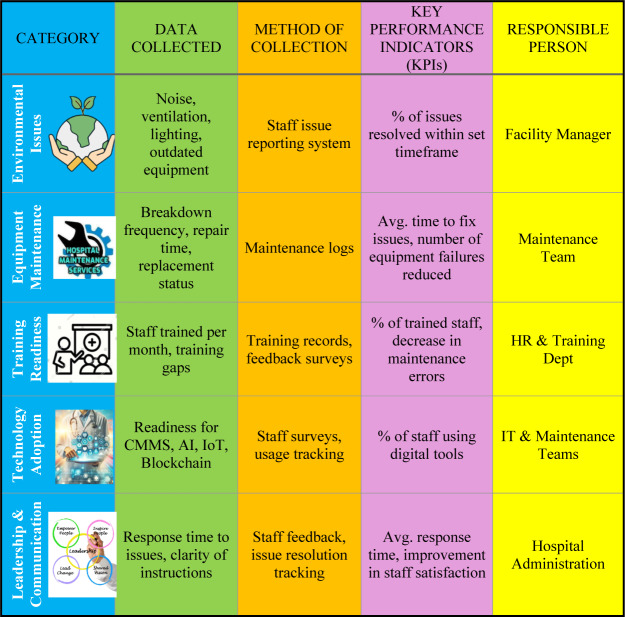

**Source(s):** Authors’ own work

1. *Implementation Tip*: Start with *manual tracking* (Excel, Google Sheets) and gradually integrate the tool into hospital IT systems.

The proposed SMF offers a structured guide for integrating technology and management best practices, helping healthcare organisations optimise maintenance operations and improve patient care.

Strategic guidelines for hospital administrators on adopting SMF are detailed in Appendix B, outlining step-by-step the key actions for piloting, evaluating, and scaling the framework. Appendix B also includes an actionable implementation guide and a visual overview of how SMF components interact to support decision-making and continuous improvement.

### 5.3 Practical tech solutions and recommendations

Building on the study’s findings, the following solutions offer healthcare personnel and administrators clear, actionable strategies to enhance maintenance management and patient safety:

1. *Technology-driven solutions:* Implement CMMS, AI, IoT, and Blockchain to streamline processes, minimise equipment downtime, and improve working conditions.
2. *Key recommendations*: The study outlines specific, easily adoptable actions for staff and administrators (see Section 7 for detailed suggestions).

By implementing these research-based strategies, healthcare facilities can improve maintenance management, boost staff satisfaction and productivity, and enhance patient safety and healthcare quality.

In summary, the practical contributions of this research include: (a) the SMART-Maintenance Framework (SMF), (b) strategic guidelines for adopting the SMF effectively, (c) technology-driven solutions about emerging technologies’ integration, and (d) key recommendations for staff and administrators.

Beyond operational benefits, the SMF can support broader societal goals, including cost savings, improved resilience, and better patient outcomes. Potential barriers (such as funding limitations, digital skill gaps, and data privacy concerns) can be addressed through phased implementation, public-private partnerships, targeted micro-training, and secure platforms aligned with healthcare data regulations.

Appendix C provides a set of practical tools to support implementation and broader strategic alignment of the SMF. Table C1 summarises how SMF aligns with key theoretical foundations. Table C2 presents benchmark-based estimates of its potential economic and operational impact to assist managerial decision-making. For policy-level integration, Table C3 outlines national governance recommendations, while Table C4 proposes societal metrics—such as QALYs and patient safety indicators—for evaluating the long-term impact of SMF in future research.

## 6. Conclusion

This study has highlighted several critical aspects of facility management in healthcare settings and their impact on staff performance, the benefits of CMMS and other cutting-edge technologies like AI, IoT and Blockchain, and the role of leadership. Here are the key conclusions drawn from the results:

### 6.1 Impact of effective maintenance management on staff performance

Effective maintenance management significantly enhances staff performance by creating a safer and more functional working environment. The study identified key environmental challenges, such as high winter humidity and noise levels, which contribute to staff stress and impact job satisfaction and productivity.

While satisfaction with building structures, lighting, and air quality is moderate, outdated and failing equipment, poor workplace design, and a lack of communication between maintenance management and staff exacerbate stress levels. The timely resolution of maintenance issues and the reduction of technological equipment failures are crucial in minimising stress and optimising performance.

These findings highlight the need for improved maintenance practices, faster responses to issues, and better communication strategies. By addressing these challenges, healthcare facilities can foster a more supportive and efficient working environment, ultimately enhancing staff performance and job satisfaction.

### 6.2 Benefits of advanced maintenance management systems

The findings highlight the benefits of CMMS and emerging technologies in healthcare maintenance. Despite widespread use of computerised scheduling, the continued reliance on manual recording reflects an ongoing shift toward comprehensive digital integration. The overwhelming preference for advanced technologies suggests that staff recognise their efficiency in streamlining maintenance operations, reducing staff burden, and minimising errors associated with manual systems. These technologies enable proactive scheduling, real-time monitoring, and predictive maintenance, ultimately reducing equipment downtime and enhancing operational efficiency.

However, gaps remain in technological upgrades and structured training. The study demonstrated a high level of acceptance and readiness among healthcare staff for adopting these innovations, reflecting a positive organisational culture toward technological advancement. To fully realise the benefits of these systems, structured training programs are essential to ensure staff can effectively utilise them, leading to improved maintenance efficiency and overall workplace effectiveness.

### 6.3 Role of leadership in maintenance management

Leadership plays a crucial role in ensuring efficient maintenance management. The study reveals that inconsistent scheduling and delayed problem resolution reflect weaknesses in supervisory practices. Although many managers prioritise safety over cost to some extent, they still need to provide more training on equipment use and enforce stricter adherence to maintenance schedules. Strategic planning, effective communication, and prompt issue resolution consistently influence staff performance and patient safety. While many respondents acknowledge interdepartmental communication, they also recognise the need for further improvement. Promptly resolving maintenance issues emerged as the most influential factor shaping employees’ perception of maintenance organisation. Strengthening leadership through better training and a proactive approach to maintenance management can significantly improve reliability and safety of healthcare facilities.

In conclusion, this research highlights the importance of effective maintenance management, the adoption of advanced technologies, and strong leadership in enhancing healthcare efficiency and optimising staff performance. Addressing the identified challenges and leveraging modern maintenance management systems can help healthcare organisations improve operational efficiency, patient safety, and staff well-being.

## 7. Recommendations

The following recommendations outline key actions for both staff and administrators, aiming to improve maintenance practices, promote safety, and support technological advancements in healthcare settings.

### 7.1 Recommendations for healthcare personnel

*Report issues promptly*: Staff members should consistently report any maintenance issues or equipment failures to the maintenance department. Early reporting can help prevent small problems from becoming major issues.

*Participate in training programs*: Whenever training programs are available, staff members should actively participate to improve their skills in handling and maintaining equipment. Staying updated with new technologies and procedures will enhance their efficiency.

*Provide feedback*: Staff should provide regular feedback on the working environment and the effectiveness of the maintenance programs. Constructive feedback can guide administrators in making necessary improvements.

*Promote a safe work environment*: Encourage a culture of safety by adhering to maintenance protocols and safety guidelines. Staff members can lead by example and support their colleagues in following best practices.

*Communicate with administration*: Maintain open lines of communication with administration regarding maintenance concerns and suggestions for improvements. Effective communication can help bridge gaps between staff and management.

*Utilise available resources*: Make use of the resources and tools provided by the healthcare unit, such as maintenance request forms or digital reporting systems. Efficient use of these resources can streamline maintenance processes.

### 7.2 Recommendations for healthcare unit administrators

*Financial support*: Ensure the maintenance department is adequately funded to handle scheduled inspections and breakdowns promptly.

*Technological upgrades*: Invest in modernising older facilities and purchasing new machinery to keep up with technological advancements.

*Comprehensive maintenance systems*: Adopt advanced maintenance management systems to improve the organisation and monitoring of maintenance activities.

*Environmental sustainability*: Invest in energy upgrades and use sustainable energy sources to reduce environmental risks and promote recycling practices.

*Infrastructure enhancement*: Improve building structures to meet staff needs.

*Lighting improvement*: Invest in better lighting solutions to enhance employee satisfaction.

*Environmental factors management*: Implement air purification and renewal systems to reduce work-related stress.

By focusing on these recommendations, healthcare facilities can improve maintenance management practices, enhance staff performance, and ensure a safer and more efficient working environment.

## 8. Limitations of the study

Despite its valuable insights, this study has limitations that should be acknowledged to contextualise the findings and guide future research:

The use of convenience sampling, with questionnaires distributed via hospital websites and nursing social media groups, may introduce selection bias. Respondents who are more digitally active may differ in their experiences or attitudes from those less engaged online. As a result, the findings may not be generalisable beyond this specific sample or to healthcare systems in other countries.

The relatively small sample size (*n* = 102) also limits representativeness across the broader healthcare workforce. A larger, more diverse sample would strengthen external validity and allow for deeper subgroup comparisons.

The exclusive use of self-reported data may introduce social desirability and recall bias, limiting the depth and accuracy of staff experience insights. Future studies could enhance reliability by triangulating survey results with objective data sources, such as maintenance logs or incident reports, and incorporating qualitative methods like interviews or focus groups to capture more nuanced perspectives.

Finally, while the proposed SMART-Maintenance Framework (SMF) offers practical value, it remains conceptual. Future research should validate the SMF through pilot testing and longitudinal studies to assess its long-term effects on maintenance operations, staff well-being, and healthcare quality.

## Supplementary Material

Data supplement 1

Data supplement 2

Data supplement 3

## Notes

The supplementary material for this article can be found online.

## References

[ref051] Alhurayess, S. and Darwish, M.K. (2012), “Analysis of energy management in hospitals”, Proceedings of the 47th International Universities Power Engineering Conference (UPEC), Uxbridge, UK, IEEE, pp. 1-4, doi: 10.1109/UPEC.2012.6398665.

[ref003] American Hospital Association (2022), Certified Healthcare Facility Manager Handbook, American Hospital Association Certification Center, available at: Link to the website

[ref004] Becerik-Gerber, B., Jazizadeh, F., Li, N. and Calis, G. (2011), “Application areas and data requirements for BIM-enabled facilities management”, Journal of Construction Engineering and Management, Vol. 138 No. 3, pp. 431-442, doi: 10.1061/(ASCE)CO.1943-7862.0000433.

[ref005] Beniacoub, F., Ntwari, F., Niyonkuru, J.P., Nyssen, M. and Van Bastelaere, S. (2021), “Evaluating a computerized maintenance management system in a low-resource setting”, Health and Technology, Vol. 11 No. 3, pp. 655-661, doi: 10.1007/s12553-021-00524-y. 33680701 PMC7914047

[ref006] Borman, W.C. and Motowidlo, S.J. (1997), “Task performance and contextual performance: the meaning for personnel selection research”, Human Performance, Vol. 10 No. 2, pp. 99-109, doi: 10.1207/s15327043hup1002_3.

[ref007] Buljac-Samardzic, M., Doekhie, K.D. and van Wijngaarden, J.D.H. (2020), “Interventions to improve team effectiveness within health care: a systematic review of the past decade”, Human Resources for Health, Vol. 18 No. 1, p. 2, doi: 10.1186/s12960-019-0411-3. 31915007 PMC6950792

[ref009] Cotts, D.G., Roper, K.O. and Payant, R.P. (2009), The Facility Management Handbook, AMACOM, New York, NY.

[ref010] Dhillon, B.S. (2002), Engineering Maintenance: A Modern Approach, CRC Press, Boca Raton.

[ref011] Duigou, A. (2023), “AI-driven predictive maintenance in industrial and healthcare settings: a review”, Journal of Maintenance Engineering, Vol. 12 No. 3, pp. 45-62.

[ref052] Fatima, T., Malik, S.A. and Shabbir, A. (2018), “Hospital healthcare service quality, patient satisfaction and loyalty: an investigation in context of private healthcare systems”, International Journal of Quality & Reliability Management, Vol. 35 No. 6, pp. 1195-1214, doi: 10.1108/IJQRM-02-2017-0031.

[ref014] Griffin, M.A., Neal, A. and Parker, S.K. (2017), “A new model of work role performance: positive behavior in uncertain and interdependent contexts”, Academy of Management Journal, Vol. 50 No. 2, pp. 327-347, doi: 10.5465/amj.2007.24634438.

[ref015] Hamed, H.A., Majeed, A.R., Abbas, M.S. and Majeed, T.R. (2025), “Comparative study of device lifecycles in different healthcare settings”, Engineering and Technology Journal, Vol. 10 No. 1, pp. 3517-3525, doi: 10.47191/etj/v10i01.09.

[ref016] HyScaler (2025), “Blockchain for facility management: ensuring data integrity and collaboration”, Industry Report, HyScaler Technologies.

[ref017] Innovent.io (2023), “IoT solutions for predictive maintenance in smart facilities”, Innovent White Paper Series, Innovent.io.

[ref019] Kaplan, R.S. and Porter, M.E. (2011), “How to solve the cost crisis in health care”, Harvard Business Review, Vol. 89 No. 9, pp. 46-52. 21939127

[ref020] Kasyapa, M.S.B. and Vanmathi, C. (2024), “Blockchain integration in healthcare: a comprehensive investigation of use cases, performance issues, and mitigation strategies”, Frontiers in Digital Health, Vol. 6, 1359858, doi: 10.3389/fdgth.2024.1359858. 38736708 PMC11082361

[ref053] Kaur, J. (2024), “AI agents empowering predictive maintenance for hospital equipment”, Akira AI, available at: Link to the website (accessed 25 February 2025).

[ref054] Kelvin-Agwu, M.T.C., Adelodun, M.O., Igwama, G.T. and Anyanwu, E.C. (2024), “The impact of regular maintenance on the longevity and performance of radiology equipment”, International Journal of Engineering Research and Development, Vol. 20 No. 9, pp. 171-177.

[ref021] Krijgsheld, M., Tummers, L.G. and Scheepers, F.E. (2022), “Job performance in healthcare: a systematic review”, BMC Health Services Research, Vol. 22 No. 1, Article 149, doi: 10.1186/s12913-021-07357-5. PMC8815187 35120495

[ref022] Laturkar, A. and Khaiyum, S. (2023), “Blockchain for healthcare: improving interoperability, data integrity, and patient privacy”, International Journal of Engineering Research and Technology, Vol. 11 No. 6, doi: 10.17577/NCRTCA-PID-014.

[ref024] Letsios, A., Polyzos, N., Poulopoulos, Ch. and Skamnakis, Ch. (2022), “Hospital managers' participation in operational planning: insights from a recent study in the Greek National Health System”, Hippokratia, Vol. 26 No. 3, pp. 91-97. 37324045 PMC10266327

[ref026] Luschi, A., Daino, G.L., Ghisalberti, G., Mezzatesta, V. and Iadanza, E. (2024), “Empowering clinical engineering and evidence-based maintenance with IoT and indoor navigation”, Future Internet, Vol. 16 No. 8, p. 263, doi: 10.3390/fi16080263.

[ref028] Mahfoud, H., Abdellah, E.B. and El Biyaali, A. (2018), “Dependability-based maintenance optimization in healthcare domain”, Journal of Quality in Maintenance Engineering, Vol. 24 No. 2, pp. 200-223, doi: 10.1108/jqme-07-2016-0029.

[ref030] Maslach, C. and Leiter, M.P. (2016), “Understanding the burnout experience: recent research and its implications for psychiatry”, World Psychiatry, Vol. 15 No. 2, pp. 103-111, doi: 10.1002/wps.20311. 27265691 PMC4911781

[ref055] Mehta, A. (2025), “Leveraging AI, Blockchain, and IoT technologies for transformative healthcare: Enhancing data security, privacy, and operational intelligence”, International Journal of Progressive Research in Engineering Management and Science, Vol. 5 No. 5, pp. 372-386.

[ref031] Mitropoulos, P., Mitropoulos, I. and Sissouras, A. (2013), “Managing for efficiency in health care: the case of Greek public hospitals”, The European Journal of Health Economics, Vol. 14 No. 6, pp. 929-938, doi: 10.1007/s10198-012-0437-0. 23111541

[ref032] Mobley, R.K. (2002), An Introduction to Predictive Maintenance, Elsevier Science, Woburn, MA.

[ref033] Ntshebe, S., Mapuranga, M., Lose, T. and Lukman, Y. (2022), “Facility maintenance management and its effects on employee performance: a positivist approach”, International Journal of Higher Education, Vol. 11 No. 7, pp. 47-54, doi: 10.5430/ijhe.v11n7p47.

[ref057] Open Medscience (2025), “AI in healthcare: Predictive maintenance and automated medical workflows”, available at: Link to the website (accessed 18 April 2025).

[ref034] Pappa, A. and Kapiki, S. (2022), “The impact of maintenance management on the working environment of health units”, Proceedings of the 3rd International Conference on Healthcare Management (ICOHEMA), Thessaloniki, Greece, 18-20 November, pp. 18-20.

[ref035] Pomaranik, W. and Kludacz-Alessandri, M. (2024), “Talent management practices and other factors affecting employee performance in the public healthcare sector in Poland: an empirical study using structural equation modelling”, BMC Health Services Research, Vol. 24 No. 1667, doi: 10.1186/s12913-024-12169-4. 39736617 PMC11687060

[ref036] Sebastian, R. (2011), “Changing roles of the clients, architects and contractors through BIM”, Engineering Construction and Architectural Management, Vol. 18 No. 2, pp. 176-187, doi: 10.1108/09699981111111148.

[ref037] Shamayleh, A., Awad, M. and Farhat, J. (2020), “IoT based predictive maintenance management of medical equipment”, Journal of Medical Systems, Vol. 44 No. 72, doi: 10.1007/s10916-020-1534-8. 32078712

[ref039] Shohet, I.M. and Lavy, S. (2004), “Healthcare facilities management: state-of-the-art review”, Facilities, Vol. 22 No. 7-8, pp. 210-220, doi: 10.1108/02632770410547570.

[ref040] Tsang, A.H.C. (1995), “Condition-based maintenance: tools and decision making”, Journal of Quality in Maintenance Engineering, Vol. 1 No. 3, pp. 3-17, doi: 10.1108/13552519510096350.

[ref041] Venkatesh, V., Morris, M.G., Davis, G.B. and Davis, F.D. (2003), “User acceptance of information technology: toward a unified view”, MIS Quarterly, Vol. 27 No. 3, pp. 425-478, doi: 10.2307/30036540.

[ref044] Wong, J.K.W., Li, H. and Wang, S.W. (2005), “Intelligent building research: a review”, Automation in Construction, Vol. 14 No. 1, pp. 143-159, doi: 10.1016/j.autcon.2004.06.001.

[ref042] Wireman, T. (2005), Total Productive Maintenance, Industrial Press, New York, NY.

[ref056] World Health Organization (2015), “Hospital Safety Index: Guide for evaluators” Geneva: WHO, available at: Link to the website (accessed 30 June 2025).

[ref058] Yousefli, Z., Nasiri, F. and Moselhi, O. (2017), “Healthcare facilities maintenance management: a literature review”, Journal of Facilities Management, Vol. 15 No. 4, pp. 352-375, doi: 10.1108/JFM-10-2016-0040.

