# Supplementary material for: Enhancing healthcare efficiency: leveraging advanced maintenance management for optimal staff performance
Source: J Health Organ Manag. 2025 Sep 9;39(9):398–418. doi: 10.1108/JHOM-03-2025-0134 (PMC12520617; doi:10.1108/JHOM-03-2025-0134)
Supplement: Data supplement 1 [file jhom-03-2025-0134_suppl1.docx]

**Appendix A. Anova Results and Comparative Charts**

## Table A1. ANOVA Results: Perceived Maintenance Burden by Job Role

| Source | SS (Sum of Squares) | df | MS (Mean Square) | F | η² |
| --- | --- | --- | --- | --- | --- |
| Between Groups | 3.33 | 4 | 0.83 | 2.88 | 0.106 |
| Within Groups | 30.00 | 105 | 0.29 |  |  |
| Total | 33.33 | 109 |  |  |  |

**Source:** Authors’ own work

## Table A2. Pearson Correlation Analysis Across Key Variables

| No. | Variable Pair | Pearson r | Strength/Direction | Interpretation |
| --- | --- | --- | --- | --- |
| 1 | **Stress Levels** vs. **Equipment Reliability** | 0.43 | Moderate Positive | Staff experiencing more frequent equipment issues tend to report higher stress |
| 2 | **Staff Satisfaction** vs. **Leadership Evaluation** | 0.53–0.62 | Strong Positive | Higher satisfaction correlates with stronger trust in leadership’s safety and decision-making |
| 3 | **Training Received** vs. **Maintenance Delays** | ~–0.34 | Moderate Negative | The more training staff receive, the less they report being burdened by delayed maintenance |
| 4 | **Technology Readiness** vs. **Perceived Performance** | 0.43–0.55 | Moderate to Strong Positive | Units with more adaptive maintenance systems and advanced tech report higher perceived organizational performance |

**Source**: Authors’ own work

The correlation analysis reveals meaningful relationships aligned with the SMART-Maintenance Framework (SMF). A moderate positive correlation (r = 0.43) between stress levels and equipment reliability indicates that frequent technical malfunctions and delayed maintenance contribute to elevated staff stress, regardless of the respondent's role. Implication: Streamlining equipment maintenance may alleviate staff burnout at an organizational level, not just among technical staff.

A strong positive correlation (r = 0.53–0.62) links staff satisfaction with leadership evaluation, underscoring how perceptions of managerial responsiveness, particularly in handling safety and setting priorities, play a pivotal role in shaping overall morale. Implication: Investment in transparent leadership and safety-focused culture can uplift performance broadly.

Moreover, a moderate negative correlation (~–0.34) between training and maintenance delays suggests that better-trained staff experience fewer disruptions. Implication: Consistent practical training equips staff to manage or even anticipate system breakdowns, strengthening resilience throughout the organization.

Finally, moderate to strong positive correlations (r = 0.43–0.55) between technology readiness and perceived performance demonstrate that health units embracing advanced systems and planned maintenance report higher efficiency and coordination. Implication: Readiness is not just about infrastructure—it’s about operational agility. Investing in advanced tools and proactive scheduling enhances the performance perception among all staff groups.

These findings provide early empirical support for the SMF’s core assumptions and offer a foundation for future causal validation.

- This bar chart illustrates the mean perceived burden score due to maintenance delays for nurses and doctors. The score scale ranges from 1 (“No burden”), 2 (“Somewhat”), to 3 (“Yes, significant burden”). Doctors report a higher average burden than nurses, indicating a statistically significant difference.

## Figure A1. Perceived Maintenance Burden – Nurses vs Doctors

## Source: Authors’ own work

Doctors reported a significantly higher maintenance burden than nurses, likely due to their greater reliance on complex diagnostic and treatment technologies, which are more sensitive to maintenance issues. Unlike nurses, who often use standardised instruments, doctors frequently depend on advanced diagnostic equipment, imaging tools, and high-precision instruments. Delays or failures in these systems can directly disrupt clinical workflows and decision-making, leading to increased stress and a heightened sense of burden among medical staff. This supports the SMF's emphasis on the relationship between equipment reliability and staff performance, highlighting the socio-technical dynamics where technical constraints directly impact clinical roles.

- This horizontal bar chart shows the average stress level reported for various maintenance-related factors, ranked from most to least stressful.


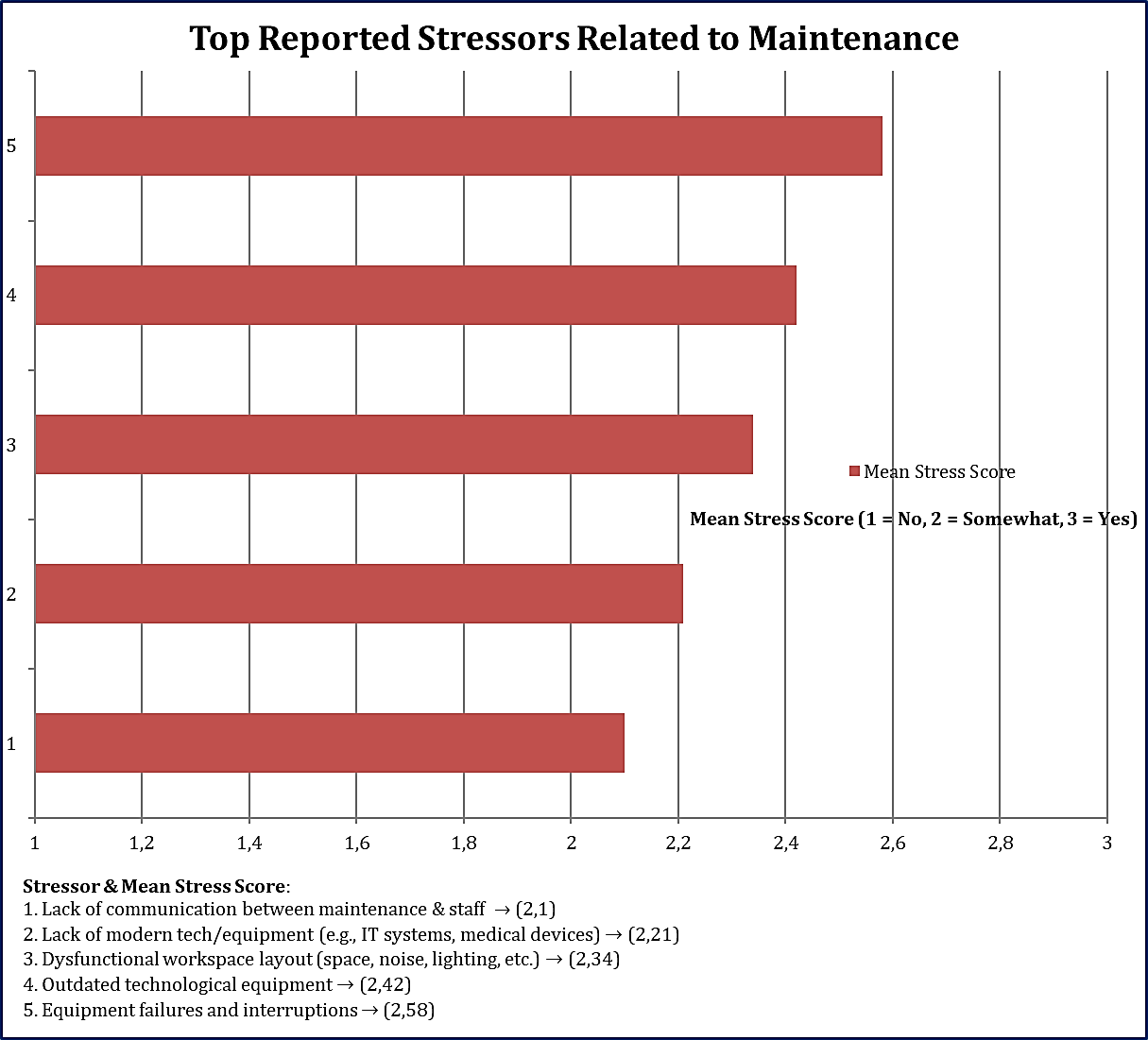


## Figure A2. Top Reported Stressors Related to Maintenance

## Source: Authors’ own work
