# Supplementary material for: Enhancing healthcare efficiency: leveraging advanced maintenance management for optimal staff performance
Source: J Health Organ Manag. 2025 Sep 9;39(9):398–418. doi: 10.1108/JHOM-03-2025-0134 (PMC12520617; doi:10.1108/JHOM-03-2025-0134)
Supplement: Data supplement 2 [file jhom-03-2025-0134_suppl2.docx]

**Appendix B. Strategic and Operational Guidance for Implementing the SMART-Maintenance Framework (SMF)**

1. **Strategic Guidelines for Hospital Administrators on Adopting SMF**

**📌** *Step 1: Secure leadership support*

- Present the business case for SMF (cost savings, improved efficiency, staff satisfaction).
- Designate a Maintenance Coordinator to oversee SMF implementation.
- Ensure alignment with hospital policies and IT infrastructure.

**📌** *Step 2: Implement a pilot program*

- Choose one hospital department (e.g., radiology, emergency) to test the framework.
- Train key personnel (maintenance teams, facility managers, IT support).
- Run the pilot for 3–6 months and collect initial feedback.

**📌** *Step 3: Leverage simple digital tools*

- Start with Google Forms/Sheets for data tracking.
- Gradually integrate with existing CMMS or hospital IT systems.
- Provide regular reports on maintenance efficiency improvements.

**📌** *Step 4: Monitor performance and adjust*

- Set up a monthly review of KPIs (issue resolution times, training rates, technology adoption).
- Gather feedback from staff and maintenance teams.
- Adapt the framework based on practical hospital needs.

**📌** *Step 5: Scale up hospital-wide*

- If the pilot is successful, expand to all hospital departments.
- Provide continuous training and support for staff.
- Establish a dedicated maintenance oversight team to sustain SMF improvements and drive continuous optimisation.

1. **SMF Implementation Guide**

The following steps outline how a hospital can adopt and implement the SMART-Maintenance Framework (SMF) *in practice*:

| 1. ****Set up a project team**** Assign staff from maintenance, IT, HR, and clinical departments. |
| --- |
| 1. ****Define KPIs and data needs**** Identify what will be monitored (e.g., noise levels, training rates, equipment failures). |
| 1. ****Develop simple tracking tools**** Create a spreadsheet or digital form to record environmental, technical, and leadership data. |
| 1. ****Start environmental and equipment monitoring**** Regularly log issues like noise, lighting, outdated devices, or space constraints. |
| 1. ****Conduct staff performance and satisfaction assessments**** Use short surveys or feedback forms to collect perceptions of maintenance impact. |
| 1. ****Review leadership response and coordination**** Track how quickly and effectively management handles maintenance problems. |
| 1. ****Evaluate results**** Compare data against KPIs to decide if action is needed. |
| 1. ****Take action based on evaluation**** If issues persist, implement system improvements (repairs, upgrades, training). |
| 1. ****Launch training plans**** Provide targeted staff training on new technologies or procedures (e.g., CMMS, AI, IoT). |
| 1. ****Repeat the evaluation regularly**** Use the same framework quarterly or biannually for continuous improvement. |

1. **Visual Representation of the SMART-Maintenance Framework (SMF)**

The diagram below illustrates the logic and structure of the SMART-Maintenance Framework. It shows how five key domains—environmental conditions, equipment modernisation, training readiness, technology adoption, and leadership responsiveness—feed into an evaluation process that supports targeted implementation and continuous monitoring in healthcare settings. KPI outcomes from this process inform implementation decisions and guide ongoing monitoring, enabling continuous improvement in healthcare settings.

**Figure B1. Visual representation of the SMART-Maintenance Framework (SMF)**

**Source**: Authors’ own work
