# Supplementary material for: Enhancing healthcare efficiency: leveraging advanced maintenance management for optimal staff performance
Source: J Health Organ Manag. 2025 Sep 9;39(9):398–418. doi: 10.1108/JHOM-03-2025-0134 (PMC12520617; doi:10.1108/JHOM-03-2025-0134)
Supplement: Data supplement 3 [file jhom-03-2025-0134_suppl3.docx]

**Appendix C. Strategic Foundations and Impact Pathways of the SMART-Maintenance Framework (SMF)**

The following tables provide theoretical, managerial, policy, and societal perspectives to support comprehensive understanding and implementation of the SMART-Maintenance Framework (SMF).

**Table C1.** Theory-to-Practice Mapping of SMF Through Core Organizational Frameworks

| Theoretical Lens | Core Principle | How SMF Operationalises It |
| --- | --- | --- |
| Socio-Technical Systems Theory | Align technology and human factors for optimal performance | SMF integrates tech (CMMS, AI, IoT, Blockchain) with staff training, workflows, and leadership to ensure both systems are jointly optimised. |
| Organizational Support Theory | Perceived managerial support influences satisfaction and performance | SMF includes leadership responsiveness and communication as core indicators, improving trust and morale. |
| Resource-Based View (RBV) | Internal resources (people, systems) provide competitive advantage | SMF treats skilled staff and reliable equipment as strategic assets monitored through KPIs. |

**Source**: Authors’ own work

**Table C2.** Economic and Operational Benefits of SMF Adoption Based on Sector Benchmarks

| Area of Impact | Benchmark Source | Estimated Benefit/Impact |
| --- | --- | --- |
| CMMS Implementation | Riddle Hospital (USA) | 30% reduction in service calls, 15% improvement in staff productivity |
| Downtime Reduction (via IoT/AI) | Singapore General Hospital (2024) | 25% decrease in equipment downtime |
| Staff Turnover & Stress Costs | Maslach & Leiter (2016); WHO (2014) | Reduced burnout lowers turnover costs by €1,500 – €3,000 per employee/year |
| Maintenance-Related Errors | Becerik-Gerber *et al.* (2011) | Improved scheduling reduces emergency maintenance costs by 20% |
| Blockchain for Maintenance Records | Kasyapa & Vanmathi (2024) | Improved data security & auditability and reduced administrative reconciliation time by 20–30%, with estimated annual savings of €8,000 – €12,000 in mid-sized facilities. |

**Source:** Authors’ own work

**Table C3**. Strategic Policy Recommendations for National Healthcare Maintenance Reform

| Policy Area | Recommendation |
| --- | --- |
| National Maintenance Standards | Establish minimum CMMS use across all Greek NHS units to ensure standardised maintenance tracking and reporting. |
| Digital KPI Mandates | Require public hospitals to report on digital maintenance KPIs (e.g., response time, downtime, tech adoption rates). |
| Training & Certification | Introduce national maintenance training programs with optional certification for technical and leadership staff. |
| Funding Incentives | Provide subsidies or performance-based funding to hospitals adopting advanced maintenance systems. |

**Source:** Authors’ own work

**Table C4**. Long-Term Societal Metrics for Evaluating the Impact of SMF Implementation

| Metric Type | Example Application |
| --- | --- |
| Patient Safety Outcomes | Reduction in adverse events linked to equipment failure or delayed maintenance |
| Quality-Adjusted Life Years | Increased QALYs due to improved equipment reliability and monitoring in critical care |
| Staff Retention & Wellbeing | Decreased burnout and turnover, leading to more consistent and experienced care delivery |
| Public Satisfaction | Enhanced trust and satisfaction with public healthcare services through better outcomes. |

**Source:** Authors’ own work
